# Case in Which the Arteria Innominata Was Tied by a Surgical Operation

**Published:** 1819-07-01

**Authors:** Valentine Mott

**Affiliations:** Professor of Surgery in the University of New York, &c.


*Case in which the Arteria Innominata was tied by a Surgical Operation.* By VALENTINE MOTT, M.D. Professor of Surgery in the University of New York, &c.

THROUGH the kindness of our friend and correspondent, Dr. Thomas Seeds of New York, we have been favoured with Dr. Mott's full account of this interesting case, illustrated by plates of the operation, and of the appearances on dissection.

CASE. Michael Bateman, ætat. 57, a seaman, was admitted into the New York Hospital on the 1st of March 1818, for a catarrhal affection, having his right arm and shoulder much swollen. After the subsidence of the catarrh and of the general tumefaction, an irregular tumour shewed itself, above and posterior to the clavicle. He stated that, about a week before entering the hospital, he had a fall on ship-board, from which he felt but little inconvenience at the time. Two days subsequently pain took place in the shoulder, with inability to lie on that side; the whole arm and shoulder then began to swell, and he was forced to give up work. Although the tumour gradually subsided by blistering, &c.; a faint and obscure pulsation, at length, was perceived, but the disease could not be pronounced aneurism. On the 3d of May, the patient observed that he felt something give way in the tumour, which now suddenly increased in size, and the pulsation became distinctly perceptible. By the 10th of the same month, the tumour had risen full an inch above the clavicle.

*Operation, May* 11*th.* Seventy drops of laudanum were given the patient an hour before the operation. Dr. Mott commenced his incision upon the tumour, just above the clavicle, and carried close to the bone and upper end of the sternum, terminating it immediately over the trachea, its extent being about three inches. Another incision, same length, was carried from the termination of the first, along the inner edge of the sterno-cleido mastoid muscle. The integuments were then dissected from the platisma myoides, and turned up over the tumour and neck. Cutting through the pl. myoid. Dr. M. cautiously divided the sternal portion of the mastoid muscle, and as much of the clavicular portion, as the size of the swelling would permit. The internal jugular somewhat embarrassed this part of the operation. The muscle, however, was separated as far as possible, to make room for the subsequent steps. The sterno-hyoideus was next divided, and then the sterno-thyroid turned up on the opposite side of the wound, over the trachea. This exposed the sheath of the carotid, jugular and par vagum. A little above the sternum he exposed the carotid, and separated the par vagum from it; then drawing the nerve and vein to the outside, and the artery towards the trachea, he readily laid bare the subclavian about half an inch from its origin. This was done principally by the handle of the scalpel. On arriving at the subclavian, it appeared enlarged, and of an unhealthy colour; it was, therefore, deemed injudicious by Drs. Mott, Port, Kissam, and Stevens, to pass a ligature round it. It was therefore determined to tie the innominata. The bifurcation of this vessel was already in view, and it only remained to prosecute the dissection a little lower behind the sternum, which was done principally with a knife, blunt at the sides, and sharp at the point; an instrument which Dr. M. strongly recommends in all operations about these parts. He took care to carry his dissection directly over and along the upper surface of the artery. After fairly denuding the vessel above, the cellular substance was cautiously detached from its sides, without wounding the pleura. A round silken ligature was now readily passed around it, and the artery was tied about half an inch below the bifurcation. The recurrent and phrenic nerves were not disturbed. The noose was drawn gradually, so as to ascertain the effect on the heart and lungs; but as no inconvenience seemed to result, the knot was completely secured; when the pulsation in the arm and neck ceased, and the tumour shrunk one third in size. The patient replied to interrogation that he felt no unusual sensation. The parts were brought into coaptation, and the integuments secured by three interrupted sutures with adhesive strap, &c. Three small arteries were tied in the course of the operation, and the patient lost from two to four ounces of blood. The operation itself occupied about an hour.

Ten minutes after the operation, the pulse was regular at 69; the patient perfectly comfortable; the temperature of the right arm a little decreased; breathing quite free. 2, P. M. Patient expresses a desire for food, and some soup was given him. Breathing natural, pulse as before. 3, P. M. The temperature of the arm a little more fallen; to be wrapped in cotton wadding. No unpleasant symptom. 6, P. M. Some head-ache; temperature of both arms the same; pulse a little accelerated, and a little fuller. 9, P. M. Head-ache; skin hotter than natural; pulse strong and full; carotid, on the left side dilated, and in strong action. Venesection to xvj; pulse fell 7 beats; thirst. 12, P. M. The patient has slept a little; is free from pain; pulse 60; skin moist, and of natural temperature.

*Second day.* 2, A. M. Enjoys natural undisturbed sleep; respiration free. 5, A. M. Slept well the last three hours; complains of slight head-ache, and pain in the right elbow; pulse full; skin natural and moist. To drink cream of tartar water. 10, A. M. Symptoms the same. The veins of the right arm distended; and the circulation evidently going on with considerable vigour. The radial artery is full, but not pulsating. 3, P. M. Has taken some dinner. Head-ache; pulse becomes tense, and quicker; skin hot; tongue foul. V. S. to ℥viij. Drink as before. 10, P. M. The febrile symptoms subsided; says he lies perfectly easy. A saline cathartic.

*Third day.* 5, A. M. Did not sleep, in consequence of the operation of the medicine; had several loose stools. 9, A. M. Feels comfortable; has slept a little; size of the tumour considerably diminished. The *right* facial and anterior temporal arteries pulsate distinctly. The patient slept many hours this day, and was left comfortable at midnight.

*Fourth day.* Suppuration appears through the dressings, with some fœtor; pulse soft and regular at 105. Continues comfortable, and complains of nothing.

*Fifth day.* The wound dressed to-day, and looks well. The patient is comfortable, and has no pain, or unnatural sensation; pulse 110 and strong.

*Sixth day.* Much the same; the wound granulating; distinct pulsation in the branches of the right carotid.

*Seventh day.* No alteration.

*Eighth day.* The patient feels some difficulty in swallowing; the attempt at which brings on a fit of coughing, with pain in the wound. Pulsation of the right radial artery pretty distinct.

*Ninth day.* An unexpected and alarming hæmorrhage this day took place. Dry lint, and pressure soon arrested the flow of blood.

*Fourteenth day.* The large ligature came away to day, and the wound looks perfectly healthy; his health improving in all respects. Walks about the wards.

*Twentieth day.* Has improved greatly since last report. Walked several times across the yard to day, and seemed delighted with the performance.

*Twenty-third day.* Weary of always lying on his left side, he turned over on the right, which he had not done since the operation. A gush of blood followed, and twenty-four ounces were quickly lost. Pulsation ceased in the left arm, and the patient lay gasping for breath, some minutes. The dressings were torn off, dry lint crowded into the wound, and the hæmorhage stopped.

*Twenty-fourth day.* Slept the greater part of the night, and feels comfortable, but is languid, and disinclined to eat. Wound looks pale, and the discharge is thin, and fœtid A few minutes after nine this evening, the hæmorrhage returned, and he lost about four ounces of blood before it could be stopped.

*Twenty-fifth day.* His countenance has altered for the worse; eyes heavy, and nearly fixed; cheeks sunken; tremors in all the limbs.

*Twenty-sixth day.* Continues sinking. At six in the evening, a hæmorrhage of eight ounces; after which he quietly expired.

*Examination.* The arch of the aorta and origin of the arteria innominata being fairly exposed, not a vestige of inflammation or its consequences could be discovered either upon them, the lungs, or the pleura, at any part. An incision being made longitudinally into the aorta, opposite the origin of the innominata, and upon introducing a probe cautiously up the latter vessel, it was seen to pass into the cavity of the ulcer. The innominata was then laid open with a pair of scissars into the ulcer. The internal coat of this vessel was smooth and natural about its origin, but for half an inch below where the ligature had cut through the artery, it shewed appearances of inflammation, and there was a coagulum adhering with considerable firmness to one of its sides, shewing that Nature had made an effort to plug up the extremity of so large a vessel, after the adhesion effected by the ligature had been swept away by the destructive power of ulceration. The tripod of great vessels—the innominata, subclavian, and carotid arteries, for nearly an inch, was dissolved and carried away by the ulceration. The upper surface of the pleura was thickened by the deposit of newly organized matter, for the protection of the cavity of the thorax. The internal surface of the carotid artery was lined with a coagulum of blood; the subclavian artery, internally and externally to the disease, was pervious. The brachial and other arteries of the right arm were of their natural diameter, and in every respect natural. The external thoracic or mammary arteries, as they went off from the subclavian, were larger than natural. On opening the tumour, the clavicle was found involved in it, and carious.

---

It is evident from the foregoing detail, that the practicability of tying the innominata, and the compatability of the ligature of it with life, are completely established. The *propriety* of the operation, in the individual case, has been doubted, but the principle is, at all events, confirmed. Editor.

